# Supplementary material for: Impact of Moderate Sodium Restriction and Hydrochlorothiazide on Iodine Excretion in Diabetic Kidney Disease: Data from a Randomized Cross-Over Trial
Source: Nutrients. 2019 Sep 12;11(9):2204. doi: 10.3390/nu11092204 (PMC6770176; doi:10.3390/nu11092204)
Supplement: Supplementary file 1 [file nutrients-11-02204-s001.pdf]

Supplemental table 1. List of food products and their sodium content that was given to every patient at the time of inclusion. For the periods on sodium restriction, patients were advised not to add any salt to their food and to replace sodium-rich products with sodium-poor products.

Food product	Quantity	Sodium content (mg)
**Bread**		
Rusk/cracker	1 piece	25
Roll (wheat, whole grain and white)	1 piece	300
Bread (wheat, whole grain and white)	1 slice	200
Rye bread	1 slice	200
Crisp bread (wheat)	1 slice	50
Raisin bread	1 slice	100
Matzo	1 piece	0
Gingerbread	1 slice	75
**Butter**		
Cooking fat, liquid	1 tablespoon	50
Cooking fat, solid	1 tablespoon	50
Margarine	for 1 slice	0
Oil (several kinds)	1 tablespoon	0
Butter, salted	for 1 slice	25
Butter, unsalted	for 1 slice	0
**Sliced cold meats**		
Saveloy	for 1 slice	250
Fricandeau	for 1 slice	0
Bacon	for 1 slice	125
Smoked beef	for 1 slice	300
Roast beef	for 1 slice	25
Salami	for 1 slice	200
Sausage	for 1 slice	175
**Cheese**		
Boursin	for 1 small toast	50
Brie	for 1 small toast	50
Goat milk cheese	for 1 slice	75
Cheese	for 1 slice	175
Cheese (low in salt)	for 1 slice	125
Cheese (sodium poor)	for 1 slice	25
Sheep milk cheese	for 1 slice	250
Cheese spread	for 1 slice	175
**Sweet toppings and other**		
Apple syrup	for 1 slice	0
Chocolate sprinkles	for 1 slice	0
Chocolate spread	for 1 slice	0
Egg, without salt	1 piece	75
Jam	for 1 slice	0
Syrup	for 1 slice	50
Peanut butter	for 1 slice	50
Sandwich spread	for 1 slice	150
Fruit sprinkles	for 1 slice	0
**Potatoes**		
Potato croquette	1 piece	150
Mashed potatoes (without salt)	1 serving spoon	0
Fried potatoes (without salt)	1 serving spoon	0
Cooked potatoes (without salt)	1 piece	0

Fries (without salt)	1 serving spoon	25
**Vegetables (prepared without salt)**		
Canned and jarred vegetables	1 serving spoon	125
Vegetables (deepfreeze, fresh, cooked)	1 serving spoon	25
Stir-fried vegetables	1 serving spoon	125
Creamed spinach (deepfreeze)	1 serving spoon	200
Sauerkraut, cooked	1 serving spoon	175
**Meat/fish/meat substitute**		
Sausage	1 piece	700
Hamburger	1 piece	500
Herring, salt	1 piece	825
Herring, sour	1 piece	600
Chicken burger	1 piece	825
Quorn pieces	1 portion	300
Smoked sausage	1 piece	825
Roast beef	1 piece	100
Schnitzel	1 piece	500
Bacon, without salt	1 piece	50
Tofu	1 piece	0
Vegetarian burger	1 piece	550
Fish	1 piece	100
Fish fingers	3 pieces	400
Meat, without salt	1 piece	75
**Desserts**		
Fruit	1 portion	0
Ice-cream	1 scoop	25
Custard/yoghurt/porridge/pudding	1 bowl	75
**Drinks**		
Soft drink	1 glass	0
Soft drink (light)	1 glass	25
Coffee/tea	1 cup	0
Grenadine	2 table spoons	0
Milk, buttermilk, chocolate milk	1 glass	100
Mineral-/spring water	1 glass	0
Tomato juice	1 glass	375
Fruit juice	1 glass	0
**Snacks**		
Biscuit	1 piece	25
Nuts	1 table spoon	250
Cake	1 slice	75
Potato chips	1 hand	50
Chocolate (milk)	1 bar	50
Chocolate (dark)	1 bar	0
Stuffed biscuit	1 piece	150
Mars	1 bar	100
Peanuts (salted)	1 table spoon	50
Peanuts (unsalted)	1 table spoon	0
Snickers	1 bar	225
Whole meal biscuit	1 piece	50
**Other**		
Pickle	1 piece	25
Broth	1 bowl	975
Diet salt	1 tea spoon	0

Gravy powder	1 sauce spoon	400
Soy sauce, asin	1 table spoon	850
Soy sauce, manis	1 table spoon	750
Mayonnaise	1 table spoon	50
Mineral salt	1 tea spoon	250
Mustard	1 tea spoon	150
Peanut satay sauce	1 sauce spoon	175
Soup	1 bowl	1000
Meat spices	1 tea spoon	475
Tomato ketchup	1 table spoon	150
Mashed tomatoes	1 table spoon	100
Gravy	1 sauce spoon	150
Salt / sea salt	1 tea spoon	800
Minced-meat hotdog	1 piece	875
Croquette	1 piece	400
Minipizza	1 piece	675
Pancake	1 piece	150
Pizza	1 piece	2000
Sausage roll	1 piece	550
